# Activation of the PGE_2_–EP2 pathway as a potential drug target for treating eosinophilic rhinosinusitis

**DOI:** 10.3389/fimmu.2024.1409458

**Published:** 2024-07-01

**Authors:** Kyohei Horikiri, Yoshitaka Taketomi, Kenji Kondo, Tatsuya Yamasoba, Makoto Murakami

**Affiliations:** ^1^ Department of Otolaryngology and Head and Neck Surgery, Graduate School of Medicine, The University of Tokyo, Tokyo, Japan; ^2^ Laboratory of Microenvironmental and Metabolic Health Sciences, Center for Disease Biology and Integrative Medicine, Graduate School of Medicine, The University of Tokyo, Tokyo, Japan; ^3^ AMED-CREST, Japan Agency for Medical Research and Development, Tokyo, Japan

**Keywords:** drug repositioning, EP2 agonist, eosinophilic chronic rhinosinusitis, mouse model, prostaglandin E synthase

## Abstract

Current treatments of eosinophilic chronic rhinosinusitis (ECRS) involve corticosteroids with various adverse effects and costly therapies such as dupilumab, highlighting the need for improved treatments. However, because of the lack of a proper mouse ECRS model that recapitulates human ECRS, molecular mechanisms underlying this disease are incompletely understood. ECRS is often associated with aspirin-induced asthma, suggesting that dysregulation of lipid mediators in the nasal mucosa may underlie ECRS pathology. We herein found that the expression of microsomal PGE synthase-1 (encoded by *PTGES*) was significantly lower in the nasal mucosa of ECRS patients than that of non-ECRS subjects. Histological, transcriptional, and lipidomics analyses of *Ptges*-deficient mice revealed that defective PGE_2_ biosynthesis facilitated eosinophil recruitment into the nasal mucosa, elevated expression of type-2 cytokines and chemokines, and increased pro-allergic and decreased anti-allergic lipid mediators following challenges with *Aspergillus* protease and ovalbumin. A nasal spray containing agonists for the PGE_2_ receptor EP2 or EP4, including omidenepag isopropyl that has been clinically used for treatment of glaucoma, markedly reduced intranasal eosinophil infiltration in *Ptges*-deficient mice. These results suggest that the present model using *Ptges*-deficient mice is more relevant to human ECRS than are previously reported models and that eosinophilic inflammation in the nasal mucosa can be efficiently blocked by activation of the PGE_2_-EP2 pathway. Furthermore, our findings suggest that drug repositioning of omidenepag isopropyl may be useful for treatment of patients with ECRS.

## Introduction

Eosinophilic chronic rhinosinusitis (ECRS) is resistant to antibiotic treatment and surgical therapy, which are commonly used to treat non-ECRS. The main treatments currently used for ECRS are corticosteroids and dupilumab, an anti-human IL-4/13 receptor monoclonal antibody (1). However, the long-term use of corticosteroids is associated with various adverse effects. Although dupilumab is effective for treating ECRS (2, 3), its high medical cost has been cited as a problematic issue. Therefore, there is a need for a new drug seed that would contribute to treatment and/or prevention of ECRS. Moreover, toward the development of new therapeutic strategies, an advanced mouse model of ECRS that more closely recapitulates human pathology would be needed, since nasal polyps with massive eosinophilia, a feature of human ECRS, have been less commonly observed in previous mouse ECRS models reported so far (4–7).

It is known that patients with aspirin-induced asthma have a higher rate of ECRS as comorbidity (8). Oral intake of nonsteroidal anti-inflammatory drugs (NSAIDs) can result in severe asthma attacks, nasal discharge, nasal congestion, and anaphylactic-like symptoms in severe cases (9). This is most likely because inhibition of cyclooxygenases (COXs), which are key enzymes for production of various prostaglandins (PGs), by NSAIDs causes substrate shunting of arachidonic acid (AA) toward the 5-lipoxygenase (5-LOX) pathway leading to enhanced production of pro-allergic leukotrienes (LTs) such as LTB_4_ and cysteinyl LTs (cys-LTs; LTC_4_, LTD_4_, and LTE_4_). NSAIDs increase the urine level of LTE_4_, a stable end product of cys-LTs, in patients with aspirin-induced asthma (10). In addition, blockage of the COX-dependent production of PGs has exacerbating or suppressive effects on various pathological conditions depending on disease contexts. Studies using mice deficient in several PG-biosynthetic enzymes and receptors have revealed the importance of PGE_2_ in attenuation of asthma (11, 12). Downstream of the two COX isoforms COX-1 and COX-2, PGE_2_ is synthesized mainly by microsomal prostaglandin E synthase-1 (mPGES-1; encoded by *Ptges* in mice) in various tissues including the lung (12). In *Ptges*-deficient mice, the decrease in anti-allergic PGE_2_ allows excessive production of pro-allergic cys-LTs and other PGs, leading to aspirin hypersensitivity and airway inflammation similar to aspirin-induced asthma in humans (12), highlighting that mPGES-1-derived PGE_2_ plays a crucial role in maintaining lung homeostasis. Among the four PGE_2_ receptors (EP1–4), EP2 has been implicated in protection against aspirin-induced asthma. Indeed, an EP2 agonist alleviated aspirin-induced asthma symptoms in *Ptges*-deficient mice (12). Moreover, *Ptger2*-deficient mice, which lack EP2, had exaggerated airway inflammation in an antigen-challenged asthma model (13).

In humans, *PTGES* expression is lower in the nasal polyps of individuals with chronic sinusitis than in those with normal nasal mucosa (14). Patients with NSAID intolerance have mutations in the *PTGER2* gene (15). Furthermore, PGE_2_ or an EP2 agonist inhibits the production of Th2-type cytokines by cells derived from nasal polyps *in vitro* (16). However, the exact role of the mPGES-1–PGE_2_–EP2 pathway in the pathology of sinusitis, ECRS in particular, remains unclear.

In this study, by using *Ptges* ^–/–^ mice, which do not synthesize PGE_2_ due to loss of the PGE_2_ synthase mPGES-1, we have developed a new mouse model of nasal inflammation with severe epithelial hypertrophy and eosinophil infiltration that mimics the human ECRS pathology more closely than previously reported mouse models for this disease. Furthermore, we have examined whether the ECRS-like pathology in *Ptges* ^–/–^ mice could be ameliorated by pharmacological activation of the PGE_2_–EP2 pathway in this model.

## Results

### Expression of mPGES-1 and EP receptors in human nasal polyps

**Figure 1:**
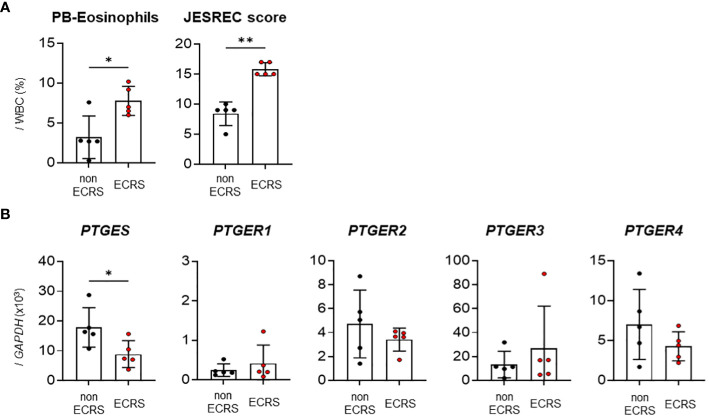
Expression analysis of PGE_2_ synthase and EP receptors in human nasal polyps. **(A)** Percentage of peripheral blood eosinophils and JESREC scores in patients with ECRS or non-ECRS. **(B)** qPCR of *PTGES* and *PTGER1–4* in human nasal polyps (n = 5). The data are expressed as the mean ± SEM, and statistical analysis was performed using Mann-Whitney U test. *p < 0.05, **p < 0.01.

First, we performed reverse transcription-quantitative polymerase chain reaction (RT-qPCR) to examine the mRNA expression levels of mPGES-1 (encoded by *PTGES*) and PGE_2_ receptors (EP1–4, encoded by *PTGER1–4*) in the nasal polyps of ECRS or non-ECRS patients. Patients with ECRS had significantly higher JESREC scores and peripheral blood eosinophil percentages (%) than patients with non-ECRS (**Figure 1A**). The nasal polyps isolated from patients with ECRS had significantly lower *PTGES* expression than those isolated from patients with non-ECRS, whereas the expression of EP receptors did not differ significantly between ECRS and non-ECRS patients (**Figure 1B**). These results raised the possibility that decreased PGE_2_ production would be involved in increased eosinophil infiltration into the nasal polyps of ECRS patients.

### *Ptges* deficiency facilitates the formation of epithelial hypertrophy with eosinophil recruitment in the nasal mucosa

**Figure 2:**
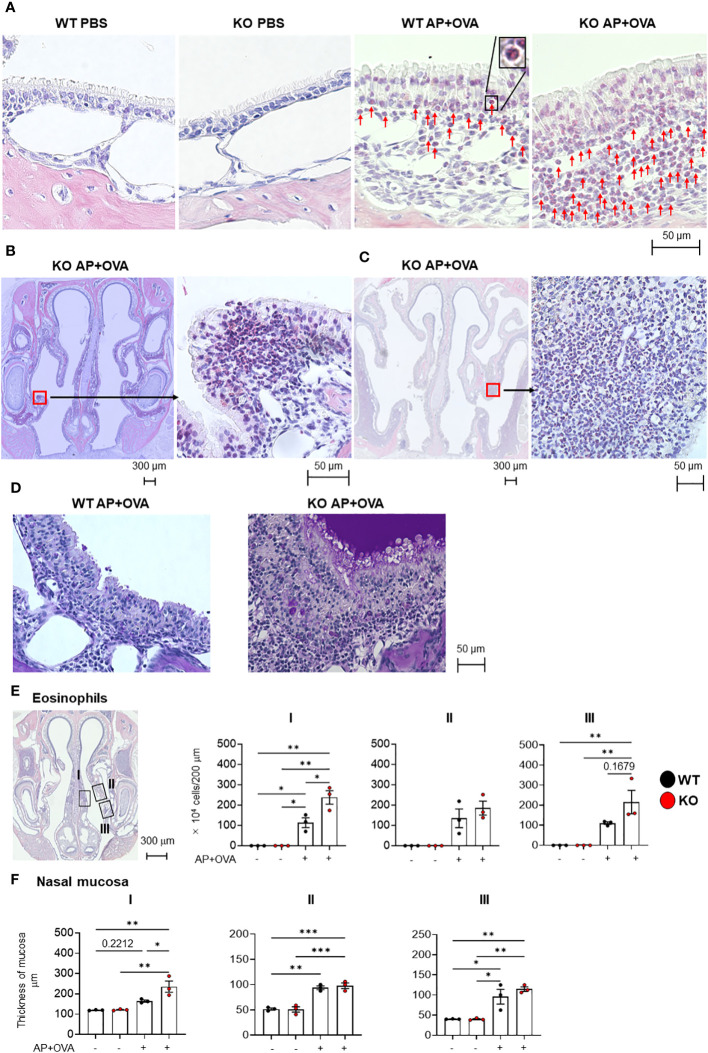
Eosinophil infiltration in the nasal mucosa of AP+OVA-challenged mice. **(A)** Nasal respiratory epithelial regions (dorsal concha) of *Ptges* ^+/+^ (WT) and *Ptges^-/-^* (KO) mice were stained with Sirius Red. Arrows indicate eosinophils. The inserted image in WT AP+OVA presents higher magnification of an eosinophil in the black box. **(B)** Sirius Red staining of the nasal respiratory epithelial region (maxillary concha) of KO mice, which had epithelial hypertrophy with massive eosinophil infiltration. **(C)** Sirius Red staining of the maxillary sinus of KO mice, which had epithelial hypertrophy with massive eosinophil infiltration. **(D)** PAS staining of the nasal respiratory epithelial region (maxillary concha) of WT and KO mice. Strongly stained mucus was observed in KO mice. **(E)** Number of eosinophils under the nasal respiratory epithelial mucosa (n = 3). Boxed regions: I: nasal septum, II: dorsal concha, III: maxillary concha. The number of eosinophils that infiltrated into a 200-μm wide area of the nasal mucosa was measured and expressed graphically as the number of cells/200 μm wide region of the nasal mucosa. **(F)** Thickness of the nasal respiratory mucosa (n = 3). The data are expressed as the mean ± SEM, and two-way ANOVA with Tukey’s multiple-comparison test was used for statistical analysis. *p < 0.05, **p < 0.01, ***p < 0.001.

To address the role of PGE_2_ in eosinophil infiltration into the nasal tissue, we took advantages of *Ptges* ^–/–^ mice. Following intranasal administration of *Aspergillus oryzae* protease (AP) and ovalbumin (OVA) to littermate *Ptges* ^+/+^ (WT) and *Ptges* ^–/–^ (KO) mice 3 times/week for 6 weeks, eosinophil accumulation was observed beneath the mucosal epithelium in both groups, which was more prominent in KO mice than in WT mice (**Figure 2A**). Neither epithelial tissue inflammation nor submucosal eosinophil infiltration was evident in phosphate-buffered saline (PBS)-treated control groups. Strikingly, epithelial hypertrophy, with massive eosinophil infiltration under the mucosa, were observed in the respiratory epithelium (**Figure 2B**) and maxillary sinus (**Figure 2C**) of AP+OVA-challenged *Ptges* KO, but not WT, mice. Periodic acid-Schiff (PAS) staining of the nasal cavity revealed that mucus production was greatly increased in KO mice compared to WT mice (**Figure 2D**). The nasal respiratory epithelium of KO mice had approximately twice as many sub-mucosal eosinophils as did WT mice in regions I (septum) and III (maxillary concha), and to a lesser extent in region II (dorsal concha) (**Figure 2E**), with the mucous layer in region I of KO mice being significantly thicker than that of WT mice (**Figure 2F**). Thus, *Ptges* deficiency promotes eosinophil recruitment into the nasal mucosa with formation of epithelial hypertrophy, which had been barely or only mildly observed in previously reported eosinophilic sinusitis models using WT C57BL/6 or BALB/c mice (**Table 1**) (4–7). Because of the severe eosinophilic inflammation with epithelial hyperplasia that extended from the respiratory epithelium (septum and maxillary concha) to the maxillary sinus, we regarded this condition as an ECRS-like model.

**Table 1:** Comparison of our present study with previous mouse ECRS models.

Reference No.	4	5	6	7	
Authors	Kim et al	Rouyar et al	Kagoya et al.	Kim et al	Present study
Mouse strain	C57BL/6	C57BL/6	BALB/c	C57BL/6	*Ptges* ^-/-^ C57BL/6
Treatment	AP + OVA	HDM + SEB	MC903 + OVA	SEB + OVA	AP + OVA
Weeks	12	21	3	12	6
Eosinophilia	+	+	+	+	++
Epithelial hypertrophy	+	–	–	+	++
Increased Th2 cytokines	+	+	+	no data	++

A summary of mouse strains used, treatment methods, administration periods (weeks), and the extents of eosinophil infiltration, epithelial hypertrophy, and increase in Th2 cytokines in past ECRS models in comparison with those in our present study. SEB (*Staphylococcus aureus* enterotoxin B), HDM (house dust mite), MC903 (a vitamin D3 analog). –, negative; +, mildly positive; ++, intensely positive.

### Increased cytokine and chemokine expression in AP+OVA-induced sinusitis model

**Figure 3:**
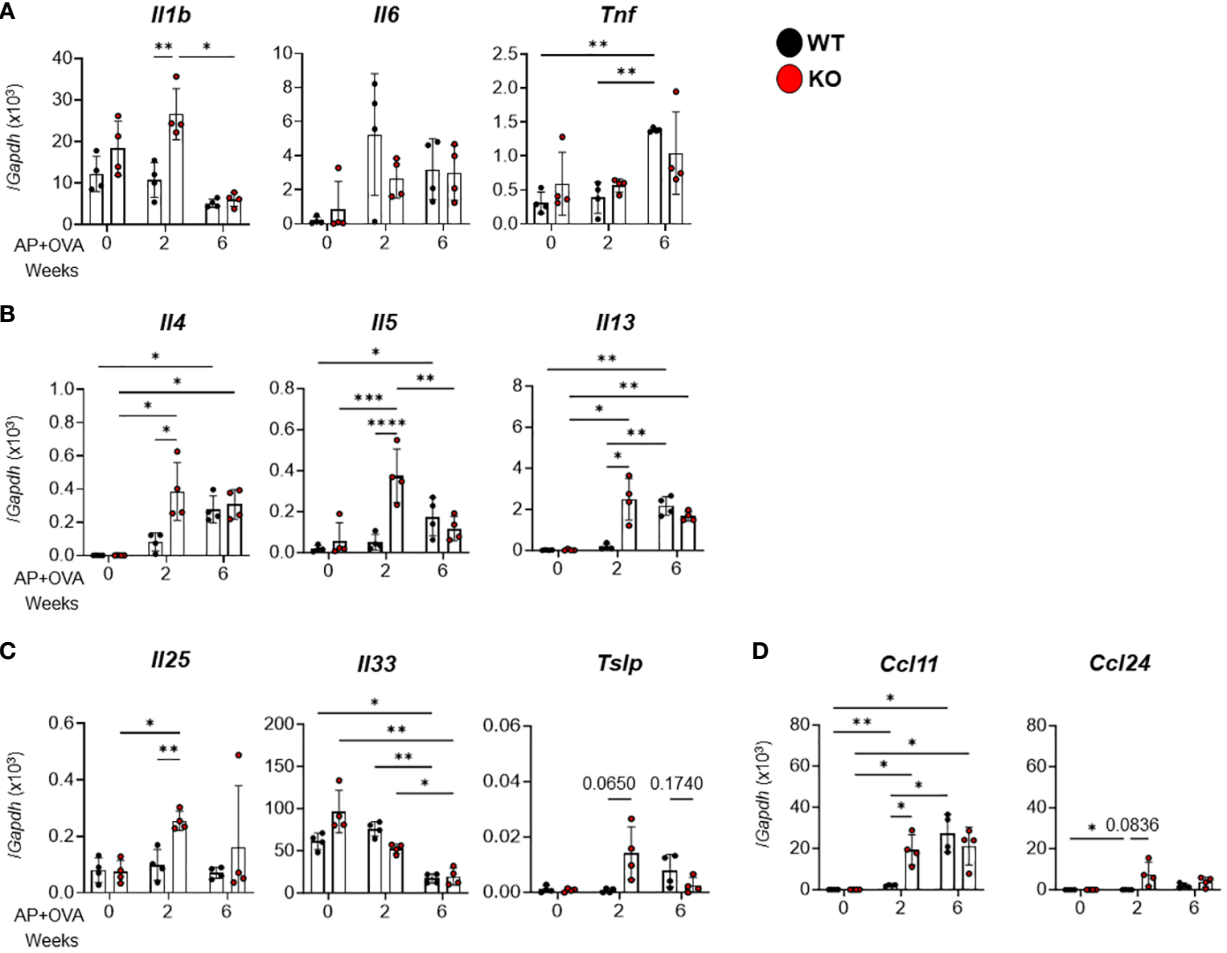
Expression of cytokines and chemokines in the nasal mucosa of AP+OVA-challenged mice. AP+OVA was administered to *Ptges* ^+/+^ (WT) and *Ptges^-/-^* (KO) mice. After isolating the nasal mucosa of the respiratory epithelium at 0, 2, and 6 weeks, qPCR of inflammatory cytokines **(A)**, Th2 cytokines **(B)**, type-2 epithelial cytokines **(C)**, and chemokines **(D)** was performed (n = 4). The data are expressed as the mean ± SEM, and two-way ANOVA with Tukey’s multiple-comparison test was used for statistical analysis. *p < 0.05, **p < 0.01, ***p < 0.001, ****p < 0.0001.

We next examined the expression levels of various cytokines and chemokines in this model by qPCR. Among the proinflammatory cytokines *Il1b, Il6* and *Tnf* (**Figure 3A**), the Th2 cytokines *Il4*, *Il5*, and *Il13* (**Figure 3B**), the type-2 epithelial cytokines *Il25, Il33* and *Tslp* (**Figure 3C**), and the eosinophil-attracting chemokines *Ccl11* and *Ccl24* (**Figure 3D**), the expression levels of *Il1b, Il4*, *Il5*, *Il13, Il25, Tslp* and *Ccl11* were significantly higher in *Ptges* KO mice than in WT mice at 2 weeks after AP+OVA challenge. Thus, the increased eosinophil accumulation in nasal mucosal tissues of KO mice may rely on the increased expression of these cytokines and chemokines involved in nasal mucosal inflammation and type-2 immunity at an early stage (2 weeks after AP+OVA challenge) of the sinusitis model.

### Evaluation of lipid mediators in AP+OVA-induced sinusitis model

**Figure 4:**
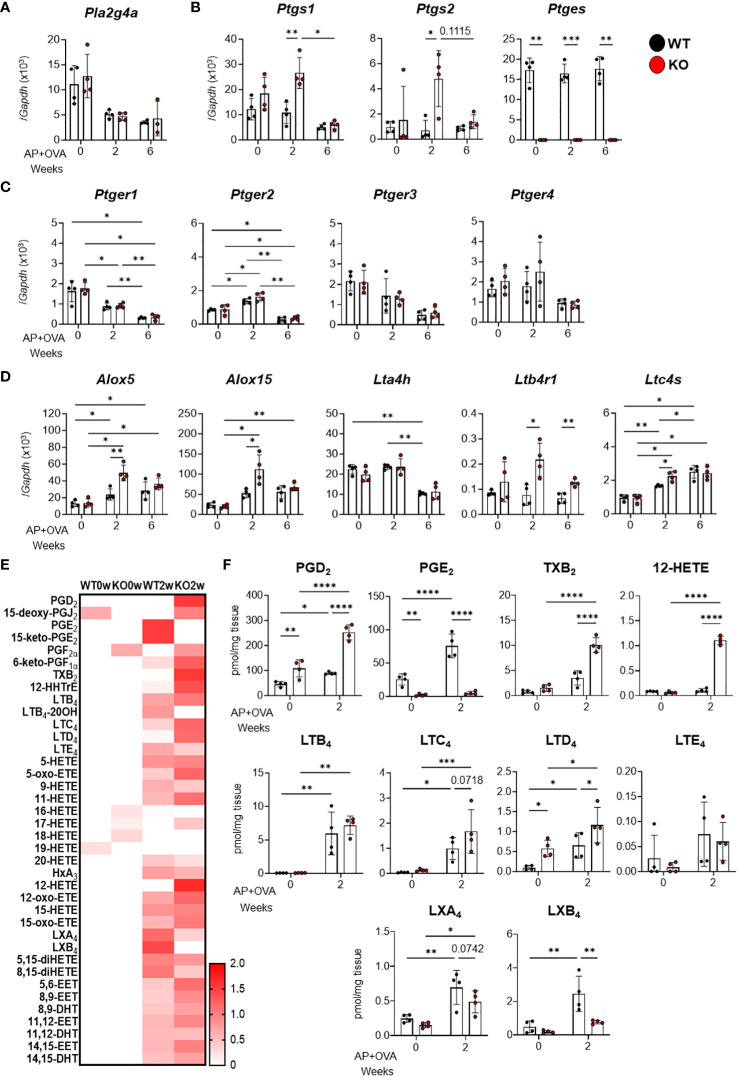
Expression of eicosanoid-biosynthetic enzymes and receptors and generation of lipid mediators in the nasal mucosa of AP+OVA-challenged mice. AP+OVA was administered to *Ptges* ^+/+^ (WT) and *Ptges^-/-^* (KO) mice. After isolating the nasal mucosa of the respiratory epithelium at 0, 2, and 6 weeks, qPCR of various biosynthetic enzymes and receptors for eicosanoids **(A-D)** and lipidomics of AA-derived lipid mediators **(E, F)** were performed (n = 4). **(A-D)** qPCR of cPLA_2_α **(A)**, PG-biosynthetic enzymes **(B)**, EP receptors **(C)**, and LT-biosynthetic enzymes and receptors **(D)**. **(E, F)** A heatmap of various AA metabolites (normalized by z-score for each metabolite) **(E)** and quantitative values of representative eicosanoids **(F)**. The data are expressed as the mean ± SEM, and two-way ANOVA with Tukey’s multiple-comparison test was used for statistical analysis. *p < 0.05, **p < 0.01, ***p < 0.001, ****p < 0.001.

Based on previous studies demonstrating that cys-LTs were overproduced in aspirin-induced asthma and that PGD_2_, thromboxane (TX) A_2_, LTB_4_, and cys-LTs promoted, while PGE_2_ inhibited, pulmonary eosinophilic inflammation (17), we analyzed the expression of eicosanoid-biosynthetic enzymes and receptors in the AP+OVA-induced ECRS-like model. Among the phospholipase *Pla2g4a* (cPLA_2_α) (**Figure 4A**), the cyclooxygenases *Ptgs1* (COX-1) and *Ptgs2* (COX-2) (**Figure 4B**), the PGE_2_ receptors *Ptger1–4* (EP1–4) (**Figure 4C**), and the LT-biosynthetic enzymes and receptors *Alox5* (5-LOX)*, Alox15* (15-LOX)*, Lta4h* (LTA_4_ hydrolase = LTB_4_ synthase), *Ltb4r1* (BLT_1_, an LTB_4_ receptor) and *Ltc4s* (LTC_4_ synthase) (**Figure 4D**), the expression levels of *Ptgs1, Ptgs2, Alox5, Alox15, Lta4h* and *Ltc4s* were significantly higher in *Ptges* KO mice than in WT mice at 2 weeks after AP+OVA challenge. *Ptges* was constantly expressed in WT mice, but not in KO mice as expected, over 6 weeks (**Figure 4B**). Thus, corroborating the changes in the expression of several cytokines and chemokines (**Figure 3**), that of several eicosanoid-biosynthetic enzymes and receptors was elevated at an early stage (2 weeks after AP+OVA challenge) of the sinusitis model in *Ptges* KO mice.

Next, we performed lipidomics analysis of the nasal mucosal tissue at 0 and 2 weeks after AP+OVA administration. Consistent with the increased expression of both COX isoforms, PGE_2_ was increased approximately three times at 2 weeks after AP+OVA treatment in WT mice, whereas it was barely detected in *Ptges* KO mice as expected (**Figures 4E, F**). Furthermore, other COX metabolites such as PGD_2_ and TXB_2_ (a stable end product of TXA_2_), as well as LOX metabolites such as LTC_4_, LTD_4_ and 12-HETE, were increased more markedly in KO mice than in WT mice at 2 weeks (**Figures 4E, F**), suggesting the shunting of AA toward other prostanoids and LOX metabolites in the absence of mPGES-1-driven PGE_2_ synthesis. Thus, in the context of lipid mediator signaling, the increased eosinophil infiltration in *Ptges* KO mice at 2 weeks after AP+OVA administration may rely, at least in part, on the increased production of pro-allergic COX (*e.g*., PGD_2_ and TXA_2_) and LOX (*e.g*., LTB_4_ and cys-LTs) metabolites in association with the decreased production of anti-allergic PGE_2_. In addition, the decrease of lipoxins (LXA_4_ and LXB_4_; AA-derived specialized pro-resolving mediators (18)) at 2 weeks in KO mice relative to WT mice (**Figures 4E, F**) might also contribute to the exacerbation of nasal inflammation caused by *Ptges* deficiency. Although various EPA (**Supplementary Figures 2A, B**) and DHA (**Supplementary Figures 2C, D**) metabolites, including the pro-resolving mediators resolvins and protectin (18), were also elevated at 2 weeks after AP+OVA challenge, their levels did not significantly differ between KO and WT mice. Overall, these findings indicate that the increased production of pro-allergic cytokines, chemokines, and lipid mediators occurred during an early phase of the sinusitis pathology in *Ptges* KO mice.

### Activation of the PGE_2_–EP2 pathway suppresses eosinophil infiltration in AP+OVA-induced sinusitis model

**Figure 5:**
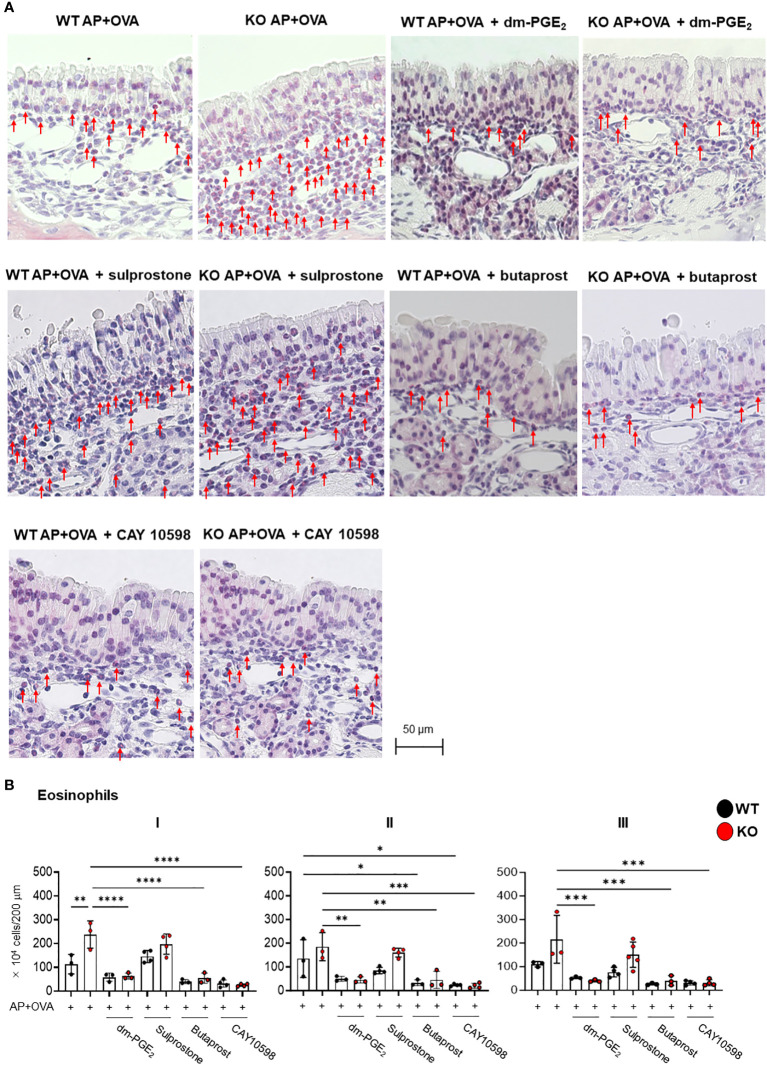
Effects of PGE_2_ or EP agonists on AP+OVA-induced sinusitis. **(A)** Sirius Red staining of the nasal respiratory epithelial region (dorsal concha) in *Ptges* ^+/+^ (WT) and *Ptges^-/-^* (KO) mice at 6 weeks after AP+OVA challenge in the presence or absence of dm-PGE_2_, sulprostone, butaprost, or CAY 10598. Arrows indicate eosinophils. **(B)** Number of eosinophils under the nasal respiratory epithelial mucosa. The data represent the mean ± SEM (n = 3–4), and two-way ANOVA with Tukey’s multiple-comparison test was used for statistical analysis. *p < 0.05, **p < 0.01, ***p < 0.001, ****p < 0.0001.

Given that the aspirin-induced, asthma-like lung response in *Ptges* KO mice is alleviated by treatment with AE1–259-01, an EP2 agonist (12), it is possible that activation of PGE_2_–EP2 signaling may also improve excessive eosinophilic inflammation in the nasal sinuses. Therefore, we investigated whether several EP agonists could prevent the ECRS-like pathology observed in *Ptges* KO mice. To this end, AP+OVA, along with either a PGE_2_ analog (16,16-dimethyl-PGE_2_; dm-PGE_2_), an EP2 agonist (butaprost), an EP1/3 agonist (sulprostone), or an EP4 agonist (CAY10598), were administered intranasally into KO and WT mice 3 times/week for 6 weeks, and eosinophil infiltration into the nasal mucosa was evaluated at 6 weeks (**Figure 5A**). Significantly fewer eosinophils were found in the nasal mucosal regions I, II, and III of the groups treated with dm-PGE_2_, butaprost, or CAY 10598 than in those of the group without treatment in KO mice and even WT mice (**Figure 5B**). In contrast, sulprostone failed to suppress eosinophil infiltration. These findings suggest that the ECRS-like pathology observed in *Ptges* KO mice is alleviated by intranasal treatment with agonists for EP2 or EP4, both of which are coupled with Gs-dependent cAMP signaling (19).

**Figure 6:**
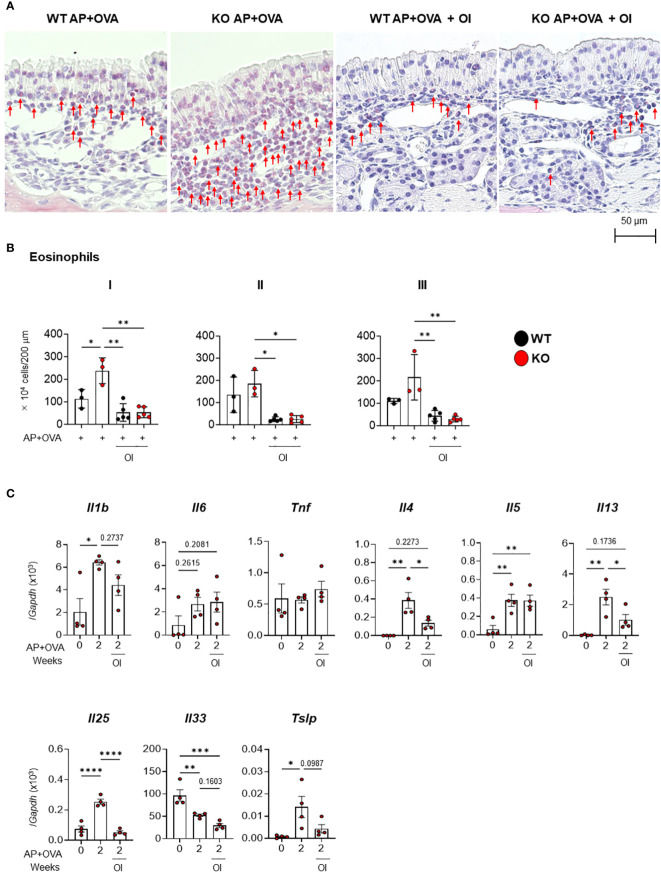
Effect of OI on AP+OVA-induced sinusitis. **(A)** Sirius Red staining of the nasal respiratory epithelial region (dorsal concha) in *Ptges* ^+/+^ (WT) and *Ptges^-/-^* (KO) mice at 6 weeks after AP+OVA challenge in the presence or absence of OI. Arrows indicate eosinophils. **(B)** Number of eosinophils under the nasal respiratory epithelial mucosa. **(C)** qPCR of cytokines in the nasal mucosa of WT and KO mice after treatment for 0 and 2 weeks with AP+OVA in the presence or absence of OI. The data represent the mean ± SEM (n = 3–4), and two-way **(B)** or one-way **(C)** ANOVA with Tukey’s multiple-comparison test was used for statistical analysis. *p < 0.05, **p < 0.01, ***p < 0.001, ****p < 0.0001.

EYBELIS ophthalmic solution with 0.002% omidenepag isopropyl (OI), an eyedrop drug used to treat glaucoma, is a clinically approved EP2 agonist in humans (20). Therefore, we next investigated whether OI treatment could prevent the ECRS-like pathology caused by AP+OVA challenge in *Ptges* KO mice. OI was intranasally administered into *Ptges* KO and WT mice along with AP+OVA 3 times/week for 6 weeks. Thereafter, we analyzed eosinophil infiltration into the nasal mucosa (**Figure 6A**). Our results showed that the group treated with AP+OVA in the presence of OI had significantly fewer eosinophils in mucosal regions I–III than did the group treated with AP+OVA alone (**Figure 6B**). Moreover, in KO mice, the increased expression of *Il4*, *Il13*, *Il25*, and *Tslp* at 2 weeks after treatment with AP+OVA was markedly suppressed by OI (**Figure 6C**). Thus, drug repositioning of OI to human ECRS is expected to lower the risk of undesirable side-effects and significantly shorten the time required for new drug development.

## Discussion

The present study has shown that an ECRS-like condition with marked eosinophil infiltration into the nasal mucosa was induced in mice lacking mPGES-1, a major PGE_2_ synthase in this tissue, following intranasal administration of AP+OVA. Notably, the period required for induction of the disease condition in AP+OVA-challenged *Ptges* KO mice was only half compared to that in several eosinophilic sinusitis models reported previously (**Table 1**) (4, 6, 7). Moreover, the present model using *Ptges* KO mice revealed the presence of epithelial hypertrophy with massive eosinophil infiltration in the nasal mucosa, which had been poorly observed in previous studies. These results suggest that mPGES-1-driven PGE_2_ plays a protective role against eosinophilic nasal polyp formation and that the use of *Ptges* KO mice offers a more accurate replication of the human ECRS pathology than the previous models.

The increased expression of type-2 cytokines and chemokines in *Ptges* KO mice relative to WT mice suggests that inflammation in the nasal mucosal epithelial tissue was exacerbated by the absence of PGE_2_. The expression levels of IL-25 and TSLP are higher in nasal polyps of patients with ECRS than in normal nasal mucosa (21, 22). Furthermore, injury to nasal mucosal epithelial cells leads to increased expression of IL-25 and TSLP, which then activate ILC2 to produce IL-5 and IL-13 (23). A similar trend toward the increased expression of these type-2 cytokines was observed in our model, suggesting that eosinophil infiltration can be accounted, at least in part, for by increased production of these cytokines, particularly IL-5 and CCL11 which directly promote eosinophil differentiation and migration. In contrast, IL-33 expression is not correlated with pathological parameters in *Ptges* KO mice, which appears to be inconsistent with the elevated expression of IL-33 in human ECRS pathology (24, 25). The effect of PGE_2_ on the expression and secretion of IL-33 in the nasal mucosa needs further elucidation.

Increased expression of *Ptgs1, Ptgs2, Alox5* and *Ltc4s*, accompanied by increased production of PGD_2_, TXA_2_ and LTs, in AP+OVA-challenged nasal mucosa in *Ptges* KO mice after 2 weeks of AP+OVA treatment suggests that mPGES-1-driven PGE_2_ puts brakes on inflammation elicited by these pro-allergic eicosanoids (26, 27). Regarding the relationship between PGE_2_ and type-2 immunity, EP2 and EP4 signals suppress the 5-LOX pathway through the Gs-dependent, cAMP–protein kinase A pathway. In aspirin-induced asthma, insufficient EP2 signaling worsens airway inflammation with increased LTB_4_ production (19). Therefore, in *Ptges* KO mice, the increased production of IL-25, TSLP, PGD_2_, and cys-LTs in the nasal mucosa may enhance ILC2 activation, which in turn stimulates the production of Th2 cytokines (IL-4, IL-5 and IL-13), leading to increased type-2 immune responses. Since the interaction of platelets with granulocytes enhances the production of TXA_2_ that plays a dominant role in pulmonary eosinophilia and vascular remodeling in the setting of PGE_2_ deficiency (28), it is likely that the increased TXA_2_ generation in the nasal mucosa by *Ptges* deficiency also exerts a similar aggravating effect on the ERCS-like pathology. In addition, PGE_2_–EP2 signaling can suppress the functions of eosinophils and other immune cells; for example, it can suppress the migration of eosinophils, decrease the production of Th2 cytokines by reducing the expression of GATA-3 (a master transcription factor for ILC2 and Th2 cells), and prevents the activation of mast cells, among others (13, 17, 29). Therefore, the decreased PGE_2_–EP2 signaling by *Ptges* deficiency may promote excessive activation of various immune cells in the nasal mucosa.

Nasal polyps are inflammatory products resulting from edematous hypertrophy of the sinus mucosa and are caused by persistent inflammation of the sinuses. Histologically, the disease primarily manifests as localized edematous swelling of the mucosa, along with inflammatory cell infiltration, myxoid degeneration, and blood vessel thinning. Nasal polyps with abnormal eosinophil buildup are seen in human ECRS. Although the detailed mechanism underlying nasal polyp formation is currently unclear, previous studies have suggested the involvement of type-2 immune responses (30, 31). ECRS is often accompanied by *Staphylococcus aureus* infection, which can exacerbate type-2 immune responses (30–32). Thus, the enhanced type-2 immune responses caused by *Ptges* deficiency may lead to greater nasal mucosal tissue damage than that occurs in WT mice, disrupting the barrier function of the mucosal surface leading to epithelial hypertrophy with severe eosinophil infiltration.

Importantly, the administration of EP2 or EP4 agonists significantly improved the ECRS-like pathology caused by *Ptges* deficiency, confirming that, as in the case of aspirin-induced asthma (12), mPGES-1-driven PGE_2_ prevents the nasal pathology via these Gs-coupled PGE_2_ receptors. From the clinical standpoint of otolaryngology, these agents can be applied topically in the form of nasal sprays. In terms of ensuring safety, local nasal sprays may cause fewer adverse effects than systemic administration and may be available at lower costs than biologics such as dupilumab. Furthermore, EYBELIS ophthalmic solution 0.002%, which contains the EP2 agonist OI, has already been approved and used in clinics as an ophthalmic drug for treatment of glaucoma. Therefore, it is expected that repositioning of this drug may significantly shorten the period required for drug development for ECRS. When administered topically as eyedrops, this drug acts on EP2 expressed in the smooth muscles of the ciliary body and trabecular meshwork within the eye (33). When administered intranasally as a nasal spray, this drug may act on EP2-expressing cells beneath the nasal mucosa with similar pharmacokinetics.

Several limitations of this study should be thoroughly considered. First, since the ethmoid sinus, where nasal polyps with large numbers of eosinophils are seen in human ECRS, does not exist in mice, we mainly analyzed the respiratory epithelium in the nasal turbinates and septum in addition to the maxillary sinus. Second, the type-2 immune response elicited in the current model was evaluated only by measuring mRNA expression levels. The spatiotemporal changes in immune cells other than eosinophils, as well as those in protein levels of cytokines and chemokines in nasal secretions and nasal mucosa, should be examined in depth to better understand the pathology of ECRS. Third, apart from stimulating the PGE_2_–EP2 pathway, no other therapeutic strategies were tested in this ECRS model. Thus, the therapeutic effect of EP2 agonists should be compared with that of drugs currently used to treat human ECRS (*e.g*., corticosteroids and dupilumab) in this model.

In conclusion, using a mouse strain that does not express the PGE_2_ synthase mPGES-1, we have generated a new mouse model of ECRS that promptly develop eosinophilia with formation of epithelial hypertrophy. Using this model, we have provided evidence that activation of the PGE_2_–EP2 (or EP4) pathway efficiently suppresses eosinophil infiltration in sinusitis. Thus, activation of this lipid signaling potentially emerges as a new strategy for treating human ECRS.

## Materials and methods

### Nasal polyps in patients

Among patients who received surgery at the Department of Otolaryngology and Head and Neck Surgery at the University of Tokyo Hospital from November 1, 2020 to December 31, 2022, we chose five patients, with ECRS who relapsed within 6 months of surgery, and five patients without ECRS, who showed improvement after surgical treatment. Patients with a JESREC score (34) of ≥11 and a nasal polyp tissue eosinophil count (400× magnification) of ≥70 were diagnosed as ECRS, and the other patients were diagnosed as non-ECRS. Analyses were performed on surgically excised nasal polyps from nasal sinuses of the patients. Oral corticosteroids were given to all ECRS patients for 1 week before surgery. Before collecting any samples, each patient provided a written informed consent. This study was authorized by the Ethics Review Board of the University of Tokyo Hospital (2020214NI).

### Mice

*Ptges* ^–/–^ mice on the C57BL/6 background were described previously (35). C57BL/6 mice were purchased from Japan SLC. Animals were housed in a specific pathogen-free room with a humidity of 50 ± 10%, a temperature of 23 ± 1°C, and a 12-h light/dark cycle (light: 8:00 to 20:00, dark: 20:00 to 8:00). The mice were provided ad libitum access to CE-2 feed (Japan SLC) and ultrafiltered water. All animal experiments were conducted in accordance with the University of Tokyo Animal Experiment Regulations (P17–032).

### Eosinophilic sinusitis model

Human ethmoid sinus, which is prone to tissue changes due to ECRS, corresponds to a part of the nasal turbinate and septum in mice. Therefore, previous reports have analyzed the respiratory epithelium of the nasal turbinates and septum as a model of sinusitis (4–7). We modified a previously described mouse model of eosinophilic sinusitis (4) by intranasally administering AP+OVA to the mice. In a protocol of 2-week treatment, 9-week-old male mice were treated with nasal spray containing 2 units of AP (Sigma-Aldrich) and 75 μg of grade-V OVA (Sigma-Aldrich) suspended in 20 μl of PBS (–) three times/week for 2 weeks. In a protocol of 6-week treatment, the same doses of AP and OVA in PBS (–) were intranasally administered to 5-week-old mice three times/week for 6 weeks. Thereafter, 0.3 mg/kg of the dm-PGE_2_ (Cayman Chemical), butaprost (Cayman Chemical), sulprostone (Abcam), or CAY 10598 (Cayman Chemical) was mixed with the AP+OVA suspension and administered intranasally as above. As required for experiments, mice were intranasally challenged with AP+ OVA in the presence of OI in EYBELIS ophthalmic solution 0.002% (Ube Industries and Santen Pharmaceutical).

### Histological analysis

After deep anesthesia, right atrial appendages of mice were incised. Thereafter, 10 ml of saline and then 10 ml of 10% neutral-buffered formalin solution (Fujifilm-Wako) were administered into the left ventricle for perfusion fixation. The heads of the mice were removed, immersed in 10% neutral-buffered formalin solution, and left at 24°C for 24 h. Thereafter, the solution was replaced with 10% EDTA-2Na solution (pH 7.0; Muto Pure Chemicals) and shaken at 24°C at 100 rpm. Finally, a neutral demineralization treatment was performed for 14 days. After decalcification, histology of the respiratory epithelium of the nasal cavity was analyzed after sectioning the head coronally, which allowed the horizontal parts of the incisor roots to be observed. Each tissue sample was placed in a Unicassette (Sakura), immersed in 70% ethanol (EtOH) for dehydration purposes, and placed in a Tissue-Tek VIP 5 Jr. tissue processor (Sakura). Thereafter, the tissue samples were immersed in paraffin, heated to 60°C using a Tissue-Tek TEC (Sakura), cooled to 4°C, and embedded. The embedded tissue was placed in a litratorome REM-710 (Yamato) and sliced to a thickness of 4 μm. To prepare the tissue sections, they were immersed in a 45°C hot bath, allowed to adhere to the slides, and then dried overnight at 45°C in a Slide Warmer (Sakura).

Eosinophils were stained with Sirius Red as described (36). Each sample preparation was transferred to a staining vat. Deparaffinization was performed twice with Xylene for 5 min, 99% EtOH for 2 min, 95% EtOH for 2 min, and 70% EtOH for 2 min. Each preparation was removed from the basket and the area around the tissue section was blocked with a water-repellent pen (Dako). Thereafter, 100 μl of Sirius Red staining solution (Muto pure chemicals) was added dropwise, and the mixture was incubated at 24°C for 1 h in a humid chamber. Subsequently, each specimen was washed with running water for 5 min, with hematoxylin for 2 min, and again with running water for 5 min. The samples were dehydrated and cleared by incubating them with 70% EtOH for 1 min, 95% EtOH for 1 min, 99% EtOH for 1 min, and xylene for 1 min twice, after which they were mounted with soft mount (Fujifilm-Wako). Tissue photos were taken using an all-in-one fluorescence microscope (BZ-X710, Keyence). Among the immune cells that infiltrated into the nasal mucosa (200-μm wide) at three locations, i.e., I: nasal septum, II: dorsal concha, and III: maxillary concha, eosinophils stained with Sirius Red were counted.

### RT-qPCR

Reagents and chemicals required for quantitative RT-qPCR were purchased from Thermo Fisher Scientific. Mice were euthanized by cervical dislocation and then decapitated, the head was sectioned sagittally from the midline, and the mucous membrane of the respiratory epithelial region of the nasal cavity was collected. TRIzol reagent (Thermo Fisher Scientific, 500 μl) was then added to the samples and homogenized with a bead homogenizer (Precellys, Bertin Instruments). Thereafter, total RNA was extracted and reverse-transcribed to cDNA using a High-Capacity cDNA Reverse Transcription Kit (Thermo Fisher Scientific). qPCR was conducted with a StepOnePlus Real-Time PCR System (Thermo Fisher Scientific) using TaqMan Gene Expression Master Mix and pre-designed primer probes (TaqMan gene Expression Assay) listed in **Table 2**, with *Gapdh* (mouse) or *GAPDH* (human) as an internal control to normalize the expression levels of individual genes.

**Table 2:** TaqMan probe assay IDs for qPCR analysis.

Gene	Assay ID
*Alox15*	Mm00507789_m1
*Alox5*	Mm01182747_m1
*Ccl11*	Mm00441238_m1
*Ccl24*	Mm00444701_m1
*Gapdh*	4352932E
*GAPDH*	4352934E
*Il13*	Mm00434204_m1
*Il1b*	Mm00434228_m1
*Il25*	Mm00499822_m1
*Il33*	Mm00505403_m1
*Il5*	Mm00439646_m1
*Il6*	Mm00446190_m1
*Lta4h*	Mm00521826_m1
*Ltb4r1*	Mm00521839_m1
*Ltc4s*	Mm00521864_m1
*Ptger1*	Mm00443098_g1
*PTGER1*	Hs00909194_g1
*Ptger2*	Mm00436051_m1
*PTGER2*	Hs00168754_m1
*Ptger3*	Mm00441045_m1
*PTGER3*	Hs00168755_m1
*Ptger4*	Mm00436053_m1
*PTGER4*	Hs00168761_m1
*Ptges*	Mm00452105_m1
*PTGES*	Hs00610420_m1
*Ptgs1*	Mm00477214_m1
*Ptgs2*	Mm00478374_m1
*Tnf*	Mm00443258_m1
*Tslp*	Mm01157588_m1

### Lipidomics

Sample extraction methods using solid-phase extraction have been previously described (37, 38). Mouse mucous membrane of the respiratory epithelial region of the nasal cavity was collected as described above. The sample was immediately frozen at -80°C, pulverized with a multi-beads shocker (YASUI KIKAI). 500 μl of methanol (MeOH) was added to the samples, sonicated for 10 min, and left at -30°C overnight. The samples were then centrifuged at 15000 x *g* for 5 min at 4°C, and the supernatant was collected. A 3 cc Oasis HLB cartridge (Waters) was used to extract polyunsaturated fatty acid metabolites. After adding 4,500 μl ultrapure water to the supernatant, the pH was adjusted to 3 with HCl and passed through the equilibrated columns. Thereafter, the column was washed with ultrapure water and hexane, and eluted with methyl formate. Finally, it was dried under nitrogen gas, dissolved in 50 μl MeOH, and used for analysis. Liquid chromatography-tandem mass spectrometry (LC-MS/MS) based lipidomics was performed on an Exion LC™ Series UHPLC coupled with a Quadrupole linear ion trap hybrid mass spectrometer (QTRAP 6500^+^) System (AB Sciex).

The extracted lipids applied to a C18 column (2.1 mm i.d. x 150 mm length, 1.7 µm particle, Phenomenex, Inc.) were separated by gradient elution with mobile phase A (water containing 0.1% acetic acid) and mobile phase B (acetonitrile:methanol, 4:1, v/v) at flow rate 0.2 mL/min at 45°C. The MS/MS analysis was performed in negative mode, and the lipids were identified by multiple reaction monitoring (MRM) transition and retention times and quantified based on the peak area of the MRM transition. The calibration curve was obtained with an authentic standard for each compound. *d4*-labeled PGE_2_ (Cayman Chemicals) and *d5*-labeled EPA (Cayman Chemicals) were added to each sample as internal standards.

### Statistical analysis

Data are expressed as mean ± standard error of the mean (SEM), and differences between two groups were determined by Mann-Whitney U test. Differences between three or more groups were determined by two-way analysis of variance (ANOVA) with Tukey’s multiple-comparison test. Statistically significant differences were defined as follows: *p < 0.05, **p < 0.01, ***p < 0.001, or ****p < 0.0001. GraphPad Prism 9 (GraphPad) was used for statistical analysis.

### Creating schematic diagrams

Schematic diagrams were created for this study using BioRender (BioRender.com).

## Data availability statement

The original contributions presented in the study are included in the article/**Supplementary Material**. Further inquiries can be directed to the corresponding author.

## Ethics statement

The studies involving humans were approved by The Ethics Review Board of the University of Tokyo Hospital. The studies were conducted in accordance with the local legislation and institutional requirements. The participants provided their written informed consent to participate in this study. The animal study was approved by The University of Tokyo Animal Experiment Regulations. The study was conducted in accordance with the local legislation and institutional requirements.

## Author contributions

KH: Writing – original draft, Writing – review & editing. YT: Writing – review & editing. KK: Writing – review & editing. TY: Writing – review & editing. MM: Writing – review & editing.
