# Supplementary material for: Activation of the PGE2–EP2 pathway as a potential drug target for treating eosinophilic rhinosinusitis
Source: Front Immunol. 2024 Jul 1;15:1409458. doi: 10.3389/fimmu.2024.1409458 (PMC11250097; doi:10.3389/fimmu.2024.1409458)
Supplement: Supplementary file 1 [file DataSheet_1.pdf]

Supplementary Material

Supplementary Figures

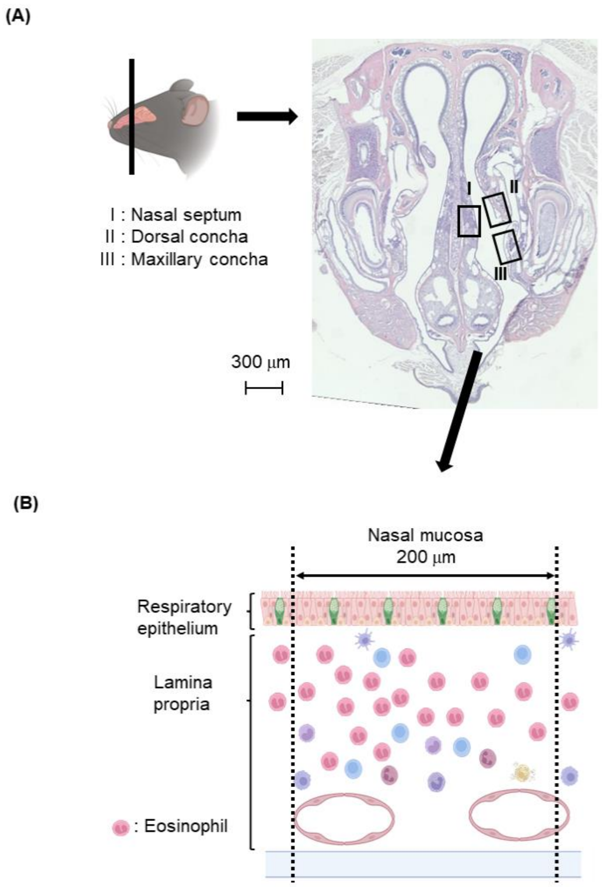

Supplementary Figure 1. Evaluation of mouse nasal mucosa. (A) Nasal respiratory epithelial region stained with Sirius Red. Boxed regions: I: nasal septum, II: dorsal concha, III: maxillary concha. (B) Schematic diagram of the nasal mucosa.

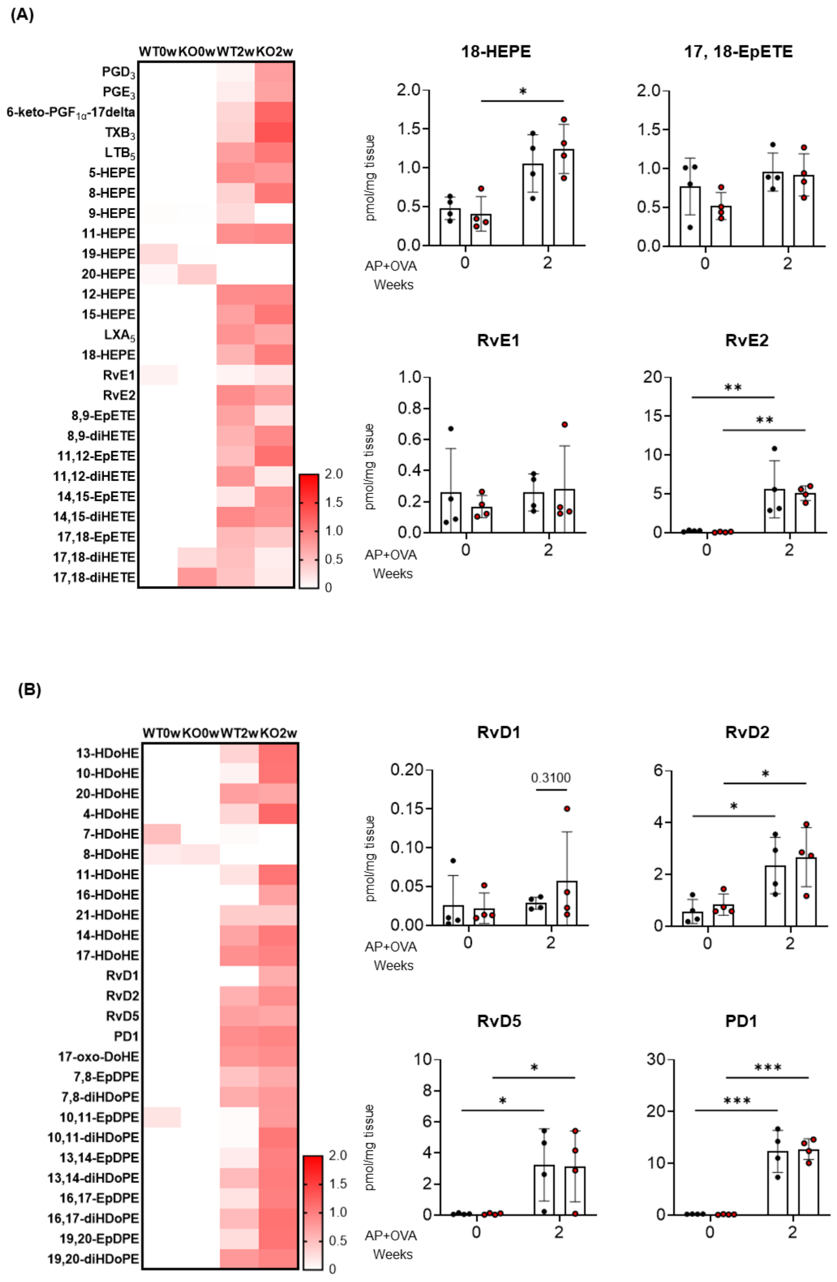

Supplementary Figure 2. Lipidomics analysis of EPA (A, B) and DHA (C, D) metabolites in the nasal mucosa of AP+OVA-challenged mice. (A, C) Heatmaps of various EPA (A) and DHA (C) metabolites (normalized by z-score for each metabolite). (B, D) Quantitative values of representative EPA (B) and DHA (D) metabolites. The data are expressed as the mean ± SEM in (B, D), and two-way ANOVA with Tukey's multiple-comparison test was used for statistical analysis. *p < 0.05, **p < 0.01, ***p < 0.001. HEPE, hydroxyeicosapentaenoic acid, EpETE, epoxyeicosatetraenoic acid; RvD, resolvin D; RvE, resolvin E; PD1, protectin D1.
